# Febuxostat and Increased Dialysis as a Treatment for Severe Tophaceous Gout in a Hemodialysis Patient

**DOI:** 10.1155/2016/9106935

**Published:** 2016-04-21

**Authors:** Lynda Ann Frassetto, Suzanne Gibson

**Affiliations:** ^1^Department of Medicine/Nephrology, University of California, San Francisco, San Francisco, CA 94143, USA; ^2^Division of Nephrology, University of California, San Francisco, San Francisco, CA 94143, USA

## Abstract

Uric acid accumulates in renal failure and is thought to be a uremic toxin—that is, higher levels of uric acid are more damaging to the kidneys. Urate crystals can precipitate in the kidney tubules, cause urate stones, and promote inflammatory changes in the renal interstitium and vascular endothelium. Uric acid is also a small non-protein-bound molecule and therefore easily dialyzable. Here, we present the case of an anuric hemodialysis patient with severe tophaceous gout who regained some renal function and whose gout burden significantly decreased resulting in marked improvement in functional status using a new gout medication, febuxostat, and increased frequency of dialysis.

## 1. Introduction

Allopurinol and colchicine have been the standard treatment for gout for decades but have significant side effects, often can not be tolerated by many patients with gout, and need to be dose adjusted in renal failure. A recently approved medication, febuxostat, selectively inhibits xanthine oxidase, the enzyme responsible for the conversion of hypoxanthine to xanthine to uric acid thereby decreasing uric acid production and blood uric acid levels. Very few side effects for febuxostat have been noted in subjects with advanced chronic kidney disease (CKD) at varying doses [1].

Uric acid has a molecular weight of 168 daltons and is water soluble and more than 95% unbound in the circulation. The clearance of uric acid is 70–80% with both low flux and high flux hemodialyzers. Three-times-a-week (TIW) hemodialysis (HD) is the standard in many western countries. Short daily hemodialysis has been shown to remove more solute (such as uric acid) than TIW hemodialysis [2].

## 2. Case Presentation

FE is a 75 y/o Caucasian man with end stage renal disease from long-standing hypertension, anuric on HD since 2012, and with severe tophaceous gout for three decades. He had no history of nephrolithiasis. He moved to California to be with his family in 2013. On arrival at our HD unit, he was wheelchair bound and unable to stand without assistance and had extremely limited mobility of his elbows, wrists, knees, and ankles due to gouty tophi, in some places > 3 × 3 cm, and soft tissue swelling. The fingers of both hands were almost completely immobilized. He was allergic to allopurinol (rash) and was on colchicine 0.6 mg/day for gout, with gout flares 2-3 times a month. He remained on colchicine throughout the subsequent treatment regimens.

His initial uric acid level at our HD unit was 10.8 mg/dL (642 *μ*mol/L). Hand X-rays demonstrated osteopenia, erosions and several lytic lesions in the radial, ulnar, and carpal bones, and large soft tissue masses, all consistent with severe bilateral gout, with no evidence of calcium pyrophosphate deposition or other arthropathies.

He was initially treated with an increase of 30 minutes per treatment in dialysis time with TIW HD for 12 hours/week, with a weekly Kt/V of 3.6 (Kt/V is a measure of dialysis adequacy; weekly minimum standard is 3.6) [3]. His uric acid levels decreased to 5.4 mg/dL (321 *μ*mol/L). His dialysis regimen was changed to four times a week (still total 12 hours/week) but he continued to have gouty attacks, though less frequently. The patient was then referred to rheumatology department for treatment options. The rheumatologist commented on the unusual severity of the gouty arthropathy [4] and recommended starting febuxostat 80 mg/day, using low dose prednisone 5–10 mg/d for flares if necessary.

His uric acid levels then decreased to below the lower limit of detection (<1.5 mg/dL [89 *μ*mol/L]) and remained there. After about three months, the patient noted that he had started to urinate again. 24-hour urine for urea and creatinine clearance showed an average glomerular filtration rate (GFR) of 3 mL/min. The patient noted that the gouty tophi were becoming smaller and softer, and he was able to move more easily, which is associated with this. Over the course of the next six months, the tophi continued to resolve. The patient was first able to get out of his wheelchair without assistance and then progressed over the next 6 months to being able to walk on his own. The incidence of gout flares decreased markedly to one every few months, treated only by increasing his colchicine dose to 0.6 mg twice a day for the duration of the flare.

More than a year after starting febuxostat, the patient has a few small tophi on his hands and left wrist. Repeat 24-hour urine for urea and creatinine clearance at this time again demonstrated an average GFR of 3 mL/min.

## 3. Discussion

To our knowledge, this is the first reported case of increasing frequency of dialysis and the use of febuxostat to improve gouty arthritis and amazingly to demonstrate some return of residual renal function in a subject who had been on hemodialysis for at least two years.

Some reports have suggested that the frequency of gouty arthritis is lower in subjects on HD compared to those with chronic kidney disease not yet on HD [5]. There are only a few reports of febuxostat use in hemodialysis patients, mostly documenting decreased serum uric acid levels, without necessarily decreasing gouty attacks [6].

The role of uric acid inducing vascular inflammation and organ damage has been of interest for some time [7]. Higher uric acid levels have been associated with increased cardiovascular and all-cause mortality in subjects with advanced CKD and those on peritoneal dialysis [8, 9].

Paradoxically, in the DOPPS trial, in countries where uric acid was commonly measured, higher uric acid levels were associated with lower mortality rates [10]. The authors suggested this was due to better nutritional status in those subjects.

Inducing higher uric acid levels in rats has been shown to induce both systemic and glomerular hypertension and macrophage infiltration in the kidney, leading to accelerated renal functional decline [11]. Several recent trials in subjects with advanced CKD have shown changes in GFR, one in subjects with asymptomatic hyperuricemia demonstrating slowing of GFR decline [12] and one demonstrating improvement in GFR after changing to febuxostat from allopurinol [13]. Increasing the frequency of dialysis is known to improve the weekly clearance of small water soluble non-protein-bound molecules like uric acid by ~20–30%. Unfortunately, Medicare, the common insurer for hemodialysis in the United States, will usually not pay for more than TIW hemodialysis unless the patient has another medical problem requiring more frequent HD (e.g., congestive heart failure).

## 4. Conclusion

To summarize the conclusions of this report

- severe tophaceous gouty arthritis is uncommon in end stage renal disease;
- febuxostat can help lower uric acid levels in subjects with CKD and on HD;
- increasing dialysis frequency, rather than length of treatment, can also help lower uric acid levels;
- together these two treatments not only lower uric acid levels but also seem to improve total body uric acid burden and can maintain or perhaps improve residual renal function, perhaps by eliminating uric acid from the kidney tissue.

## Abbreviations

CKD: Chronic kidney disease
TIW: Three times a week
HD: Hemodialysis
GFR: Glomerular filtration rate.

## Additional Points

Some of the comments from the patient more than a year after starting this combined therapy are as follows:

*It feels like a million dollars. Now I have no pain in my knees; before I couldn't walk due to pain. Now I am walking every day, and from my home to the dialysis unit. At home, I don't need any assistance. It's great.*
